# Red Light-Induced Systemic Resistance Against Root-Knot Nematode Is Mediated by a Coordinated Regulation of Salicylic Acid, Jasmonic Acid and Redox Signaling in Watermelon

**DOI:** 10.3389/fpls.2018.00899

**Published:** 2018-07-10

**Authors:** You-xin Yang, Chaoqun Wu, Golam J. Ahammed, Caijun Wu, Zemao Yang, Chunpeng Wan, Jinyin Chen

**Affiliations:** ^1^Jiangxi Key Laboratory of Crop Physiology, Ecology and Genetic Breeding, College of Agronomy, Jiangxi Agricultural University, Nanchang, China; ^2^College of Forestry, Henan University of Science and Technology, Luoyang, China; ^3^Germplasm Lab, Institute of Bast Fiber Crops, Chinese Academy of Agricultural Sciences, Changsha, China; ^4^Pingxiang University, Pingxiang, China

**Keywords:** *Citrullus lanatus*, hormones, red light, root knot nematodes, systemic resistance

## Abstract

Red light (RL) can stimulate plant defense against foliar diseases; however, its role in activation of systemic defense against root diseases remains unclear. Here, the effect of RL on root knot nematode *Meloidogyne incognita* (RKN) infestation was investigated in watermelon plants (*Citrullus lanatus* L.). Plants were exposed to 200 μmol m^-2^ s^-1^ photosynthetic photon flux density RL at the canopy level for 21 days using light-emitting photodiodes. The results showed that RL significantly suppressed gall formation and nematode development, which was closely associated with the RL-induced attenuation of oxidative stress in roots. Gene expression analysis showed that RL caused a transient upregulation of *PR1* and *WRKY70* transcripts at 7 days post inoculation in RKN-infected plants. Further investigation revealed that RL-induced systemic defense against RKN was attributed to increased jasmonic acid (JA) and salicylic acid (SA) content, and transcript levels of their biosynthetic genes in roots. Interestingly, while malondialdehyde content decreased, H_2_O_2_ accumulation increased in RL-treated RKN-plants, indicating a potential signaling role of H_2_O_2_ in mediating RL-induced systemic defense. Furthermore, analysis of enzymatic and non-enzymatic antidoxidants revealed that RL-induced enhanced defense agaist RKN was also attributed to increased activities of antioxidant enzymes as well as redox homeostasis. Taken together, these findings suggest that RL could enhance systemic resistance against RKN, which is mediated by a coordinated regulation of JA- and SA-dependent signaling, antioxidants, and redox homeostasis in watermelon plants.

## Introduction

The root-knot nematode *Meloidogyne incognita* is a soil-dwelling, microscopic nematode that feeds exclusively on the cytoplasm of living plant cells. Disease symptoms on infected plants include the presence of galls on roots, which may increase susceptibility to other pathogenic diseases such as *Fusarium wilt* (Kyndt et al., 2013). *M. incognita* invades the most economically important fruits and vegetable crops, including watermelon, tomato and cucumber and causes substantial losses around the world (Jones et al., 2013). Until now, a great deal of soil fumigants and nematicides are being used to control nematodes, which raise issues regarding food safety, environmental pollution and human health, and also threaten sustainable agricultural development. Therefore, it is indispensable to develop environmental friendly strategies of nematode control to assure food safety and sustainable crop production.

Plants have evolved various defense strategies employing intricate mechanisms that induce immune responses through complex signaling networks and molecules, including reactive oxygen species (ROS), phytohormones and defense related genes (Martìnez-Medina et al., 2017; Song et al., 2017). For instance, in response to nematode invasion, ROS were quickly accumulated in the invaded cells and cell walls, especially in the cells that are close to hypersensitive response (Melillo et al., 2006). In *Arabidopsis thaliana*, the NADPH oxidases, RbohD and RbohF, produce ROS when infected by parasitic nematodes, and thus restricting infected plant cell death and promoting nurse cell formation (Siddique et al., 2014). Plant hormones play essential role in mediating plant defense. In particular, salicylic acid (SA), jasmonic acid (JA), ethylene (ET), brassinosteroids (BRs), and abscisic acid (ABA) are key components in plant defense against nematode infection (Nahar et al., 2011; Kammerhofer et al., 2015; Kyndt et al., 2017; Song et al., 2017). Notably, JA pathway plays a pivotal role in systemic defense induction against root knot nematodes (RKN) in rice plants (Nahar et al., 2011). In tomato, JA defense-dominated genotype (35S::Prosystemin) shows stronger resistance to nematodes than that of the wild-type Castlemart and JA-deficient mutant *spr2* (Sun et al., 2010). Recent studies revealed that JA-induced enhanced defense against RKN is associated with alterations in antioxidative defense and photosynthetic processes (Bali et al., 2017). Furthermore, exogenous SA added as a soil drench is able to restrict J2s of nematodes in tomato roots by triggering a systemic acquired resistance (SAR)-like response (Molinari et al., 2014). A recent study showed that *Trichoderma* could reduce parasitic nematodes by triggering host defense, which was attributed firstly to SA-primed defense that limited root invasion of nematodes, and secondly to enhanced JA-regulated defense that antagonized the nematodes-induced deregulation of JA-dependent immunity (Martìnez-Medina et al., 2017). Interestingly, ABA interacts antagonistically with JA in rice defense against *Meloidogyne graminicola*, and thus, ABA application on rice plants aggravates nematode-caused disease symptoms (Kyndt et al., 2017). However, BRs suppress tomato defense against root-knot nematodes, without involving the classical defense pathways, such as SA, JA/ET or ABA signaling, rather triggering the apoplastic *RESPIRATORY BURST OXIDASE HOMOLOG*-dependent MPK1/2/3 activation in tomato plants (Song et al., 2017).

Light is an essential environmental signal for plant growth and development, and also plays critical role in plant defense responses to pathogens through multiple hormonal pathways and antioxidant systems (Griebel and Zeier, 2008; Roden and Ingle, 2009; Cheng et al., 2016). Light regulates plant defense mainly by modifying SA and/or JA signaling and homeostasis (Cerrudo et al., 2012; de Wit et al., 2013). For instance, plant defense against *Pseudomonas syringae* depends on the light length, while longer light length results in an elevated SA accumulation, increased transcripts of pathogenesis-related genes, and a more pronounced hypersensitive response (Griebel and Zeier, 2008). Light could suppress *P. syringae* pv. *tabaci* population in tobacco leaves through the accumulation of H_2_O_2_ during infection (Cheng et al., 2016). In addition, light quality influences plant defense against diseases by modulating response of different photoreceptor to light wavelengths. Low red/far-red ratios (R:FR) reduce *Arabidopsis* resistance to necrotrophic pathogen *Botrytis cinerea* and JA responses (Cerrudo et al., 2012). Similarly, reduced R:FR ratios inhibit both SA-dependent and JA-dependent disease resistance in *Arabidopsis* (de Wit et al., 2013).

Red light (RL) has more profound effect on activation of plant defense as compared to other monochromatic light (Wang et al., 2010; Yang et al., 2015b). For example, RL is more effective than other light qualities in inducing the defense response to powdery mildew disease through activation of SA-dependent signaling pathway, H_2_O_2_ accumulation, and associated metabolism in cucumber plants (Wang et al., 2010). This is consistent with our recent study that supplemental RL (at night) shows a more profound effect than other light quality on nematode resistance (Yang et al., 2014). However, most studies related to RL-induced resistance against pathogens were focused on above-ground plant parts, while a few studies reported RL-affected plant resistance against root-feeding nematodes. Moreover, the role of RL in SA- and JA- mediated interactions, especially in below-ground plant defense responses to root-knot nematodes is largely unknown.

Watermelon (*Citrullus lanatus*) is one of the most economically important global fruits. However, the root-knot nematode *M. incognita* causes tremendous economic losses in watermelon production throughout the world (Davis, 2007). Although light quality-induced resistance to RKN has been reported in some model plants, the role of RL in the defense response of watermelon plants to nematodes remains largely unknown. It is to be noted that the defense response of plants depends on light quality, light intensity, stress types, and plant species (Zarate et al., 2007; Hogewoning et al., 2010). Therefore, in the current study, we investigated the effect of RL on defense response to nematode infection in watermelon plants. Disease symptoms, expression of SA and JA biosynthesis-related genes, the content of SA, JA, ABA, and indole-3-acetic acid (IAA), enzymatic and non-enzymatic antioxidants were assayed. The results showed that RL exposed onto watermelon leaves could potentially activate systemic defense against RKN by modulating hormone pathways, antioxidant systems, and redox homeostasis. This study shed some lights on underlying mechanism of RL-induced systemic resistance against *M. incognita* in watermelon and may have potential implication in protected vegetable production and resistance breeding.

## Materials and Methods

### Plant Material and Growth Condition

Seeds of watermelon (*C. lanatus* cv. Xinong No. 8) were sown in trays filled with a mixture of peat and vermiculite (2:1, v/v) and placed in growth chambers at a temperature of 25/19°C (day/night), and a photoperiod of 12 hr day/night (8:00 a.m. to 8:00 p.m.), a photosynthetic photon flux density (PPFD) of 200 μmol m^-2^ s^-1^ supplied from fluorescent tubes and a relative humidity (RH) of 70%. Seedlings at the three-leaf stage were transplanted into pots filled with steam-sterilized sands and watered with Hoagland’s nutrient solution once in a week. At the four-leaf stage, seedlings were exposed to RL from 8:00 a.m. to 8:00 p.m. with a maximum wavelength at 660 nm provided by light-emitting photodiodes (LEDs, 10 W, Huizhou Kedao Technology Co. Ltd., Huizhou, China). Control plants were exposed to white LED light at the same time. The intensity of light was set at 200 μmol m^-2^ s^-1^ PPFD at the level of the canopy. Simultaneously, watermelon plants were subject to *M. incognita* infection. Mock plants that were treated with the same amount of water served as control.

The *M. incognita* (race 1) was provided by Prof. Deliang Peng, Chinese Academy of Agricultural Sciences, Beijing, China. A pure nematode culture was maintained on watermelon cultivar Xinong 8 grown in a greenhouse. Nematodes were extracted from 3-month-old infected plants using the Baermann method (Luc et al., 2005). The nematode suspension was collected after 48 h. Watermelon plants at four-leaf stage was inoculated with approximately 1000 second-stage juveniles (j2) of *M. incognita* per plant or mock treated with water pouring over the surface of the sand around the roots (Schaff et al., 2007*)*.

Thus the study comprised four treatments, such as control (mock, white light and water solution), RL (RL treatment and water solution), RKN (white light and root knot nematode *M. incognita* infection), and RL+RKN (RL treatment and root knot nematode *M. incognita* infection). Six plants served as a replicate and there were four replicates for each treatment.

Seven-days after RL and RKN treatment, leaf and root samples were harvested for biochemical and gene expression analyses. However, for defense gene expression analysis, samples are also collected at 3 dpi and 14 dpi. Immediately after harvesting, samples were frozen in liquid nitrogen and stored at -80°C. After 21 days exposure to RL and RKN, six plants from each treatment were randomly sampled for the evaluation of infection level. The roots were washed with running tap water, and the galls were counted with the aid of a stereomicroscope. Nematode susceptibility of the plants was evaluated by calculating the number of galls in the roots per plant. To visualize the galls, roots were boiled with a mixture of 0.8% acetic acid and 0.013% fuchsin for 3 min and washed with running tap water. Then the roots were destained in acid glycerol to visualize the galls.

### Determination of Malondialdehyde (MDA) and Electrolyte Leakage

The level of lipid peroxidation was estimated by quantifying the content of MDA in the roots. Root extracts were mixed with 20% trichloroacetic acid (TCA) containing 0.65% (W/V) 2-thiobarbituric acid (TBA) and incubated in boiling water for 25 min, and the reaction was stopped by immediately placing the samples in an ice bath as described previously (Hodges et al., 1999). MDA equivalents were calculated according to Hodges et al. (1999). The relative electrolyte leakage from root tissues was measured and calculated as previously described elsewhere (Cao et al., 2007).

### RNA Extraction and Quantitative Real Time PCR (qRT-PCR) Assay

Total RNA was extracted from 0.1 g of leaf and root tissues using the total RNA Miniprep Kit (Axygen Biosciences, Union City, CA, United States) according to the manufacturer’s protocol. Genomic DNA was removed with the RNeasy Mini Kit (Qiagen, Hilden, Germany). Total RNA (1 μg) was reverse-transcribed for the synthesis of cDNA using the ReverTra Ace qPCR-RT Kit (Toyobo, Japan) according to the manufacturer’s instructions. qRT-PCR was performed using the iCycler iQ^TM^ Real-time PCR Detection System (Bio-Rad, Hercules, CA, United States). The specific primers used for qRT-PCR are shown in Supplementary Table S1. PCR was performed using the SYBR Green PCR Master Mix (Takara, Tokyo, Japan). The PCR condition consisted of denaturation at 95°C for 3 min followed by 40 cycles of denaturation at 95°C for 30 s, annealing at 58°C for 30 s and extension at 72°C for 30 s. A dissociation curve was generated at the end of each PCR cycle to verify that a single product was amplified using software provided with the iCycler iQ^TM^ Real-time PCR Detection System. The software provided with the PCR system was used to calculate the threshold cycle values and to quantify the mRNA expression levels based on Livak and Schmittgen (2001). A set of multiple reference genes, clathrin adaptor complex subunit (*ClCAC*) and α-tubulin (*ClTUA*), was used as internal controls (Kong et al., 2014).

### Determination of Plant Hormones

Phytohormone extraction and quantification from watermelon leaves and roots were performed following previously described procedures with some modification (Song et al., 2017). Briefly, 100 mg of frozen leaf or root material was homogenized in 1 mL of ethyl acetate spiked with D5-JA, D5-SA, D6-IAA, and D6-ABA (OlChemIm) as internal standards to a final concentration of 100 ng mL^-1^. Tubes were centrifuged at 18,000 *g* for 10 min at 4°C. The pellet was re-extracted with 1 mL of ethyl acetate. Both supernatants were combined and evaporated to dryness under N_2_ gas. The residue was re-suspended in 0.5 mL of 70% methanol (v/v), centrifuged, and recentrifuged at 18,000 *g* for 2 min at 4°C. The supernatants were analyzed in a liquid chromatography tandem mass spectrometry system (Varian 320-MS LC/MS, Agilent Technologies, Amstelveen, Netherlands). The parent ions, daughter ions, and collision energies used in these analyses are listed in Supplementary Table S2. Phytohormone concentration was expressed as nanogram per gram of fresh mass leaf and root material.

### Determination of H_2_O_2_ Content

Content of H_2_O_2_ was determined in leaves and roots by a peroxidase (POD) assay according to Willekens et al. (1997). Plant tissues (0.3 g) were homogenized in 3 ml of HClO_4_ (1.0 M) using pre-chilled mortar and pestle. The homogenates were then transferred to 10 ml plastic tubes and centrifuged at 6000 *g* for 5 min at 4°C. Resulting supernatant’s pH was adjusted to 7.0 with 4 M KOH and centrifuged at 6000 *g* for 1 min at 4°C. The supernatant was passed through an AG1.8 prepacked column and H_2_O_2_ was eluted with double-distilled H_2_O. Equal recovery from the different samples was checked by analyzing duplicate samples. The reaction system consisted of the sample (900 μl) and 900 μl of reaction buffer containing 1 mM 2,2′-azino-di (3-ethylbenzthiazoline-6-sulfonic acid) in 100 mM potassium acetate at pH 4.4. Finally, reaction was initiated through addition of 3 μl (0.5 U) horseradish peroxidase. The absorption at 412 nm was recorded for the measurement of H_2_O_2_.

### Determination of Antioxidant Enzyme Activity, Glutathione Content, and Ascorbic Acid Content

Antioxidant enzyme activities were assayed spectrophotometrically in leaves and roots. For extraction of enzymes, frozen leaf or root sample (0.3 g) was ground with 2 mL ice-cold 50 mM PBS (pH 7.8) containing 0.2 mM EDTA, 2 mM AsA, and 2% PVP. Homogenates were centrifuged at 4°C at 12,000 *g* for 20 min, then the resulting supernatants were used for the determination of enzymatic activity. The activities of superoxide dismutase (SOD), POD, and catalase (CAT) were measured following the previously described protocols (Li et al., 2017). Ascorbate peroxidase (APX) activity was analyzed by measuring the decrease in A290 according to the method of Nakano and Asada (1981). The glutathione (GSH) content was determined according to Sgherri and Navari-Izzo (1995) by an enzymatic recycling method. Ascorbic acid (AsA) content was measured using α-α′-bipyridyl-based colorimetric assay (Gillespie and Ainsworth, 2007).

### Statistical Analysis

All data were analyzed using the statistical software SAS 8.1 (SAS Institute Inc., Cary, North Carolina, United States). The data were subjected to analysis of variance (ANOVA) and means were compared with Duncan’s multiple range test (*p* < 0.05).

## Results

### Red Light Suppresses Nematode Incidence

**FIGURE 1:**
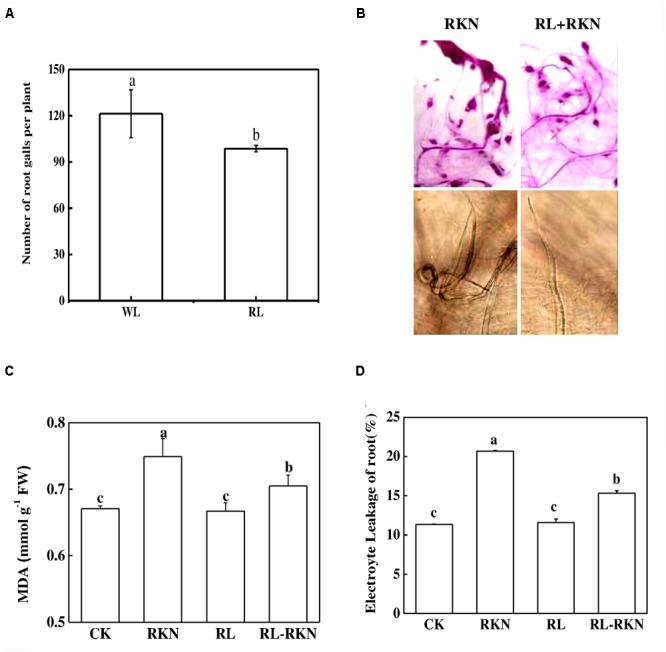
Effect of red-light on root-knot nematode-induced gall formation and oxidative stress in watermelon roots. **(A)** Numbers of galls in roots per plant under white light (WL) and red light (RL) treatment at 21 days post inoculation (dpi) with *Meloidogyne incognita*; **(B)** Root phenotype visualized by acid fuchsin staining (upper panels) and J2 nematodes inside of roots inoculated with *M. incognita* (lower panels); **(C)** Malondialdehyde (MDA) content in roots. **(D)** Electrolyte leakage in roots. Data are the means ±*SD* of four replicates. Different letters indicate statistically significant differences (Duncan’s multiple range test with a *p* < 0.05). CK, white light without *M. incognita* infection; RL, red light without nematodes infection; RKN, white light with *M. incognita* infection; RL+RNK, red light with *M. incognita* infection.

The effects of RL on the resistance of watermelon plants against nematodes were investigated by determining the gall number in the roots. The results showed that control plants had 121 galls/plant root fresh weight, in sharp contrast, RL decreased the whole gall number per plants by 19% compared with the control (**Figure 1A**). As shown in **Figure 1B**, acid fuchsin staining revealed that the galls on the roots and J2 nematodes in the roots were obviously less in RL than white light after nematodes infection. Compared with mock treatment, RKN infection significantly increased electrolyte leakage and MDA, while RL+RKN treatment remarkably reduced electrolyte leakage and MDA compared with RKN treatment (**Figures 1C,D**).

### Red Light Regulates Defense-Related Gene Expression in Leaves and Roots

**FIGURE 2:**
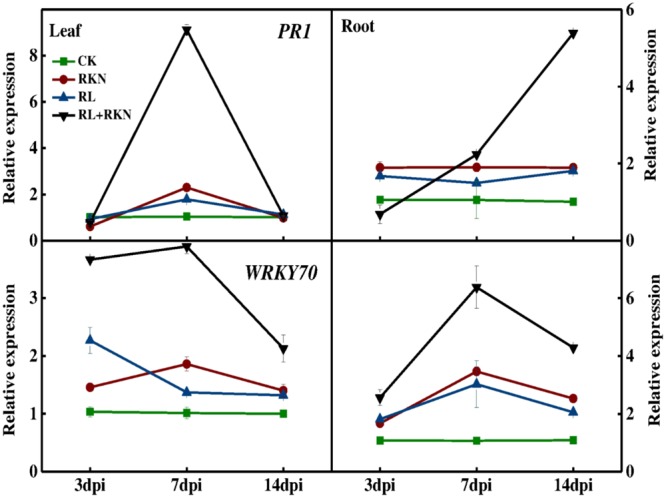
Time-course of defense related gene expression in leaves and roots of watermelon. Gene expression was assayed by qRT-PCR at 3, 7, and 14 days post inoculation (dpi). The primers used for transcript analysis are listed in Supplementary Table S1. Data are the means ±*SD* of three biological replicates. Means denoted by the same letter did not significantly differ at *p* < 0.05 according to Duncan’s multiple range test. CK, white light without *M. incognita* infection; RL, red light without nematodes infection; RKN, white light with *M. incognita* infection; RL+RKN, red light with *M. incognita* infection.

To explore the effect of RL on plant defense against nematodes, total RNA was isolated from roots and leaves at 3-, 7-, and 14 days- post inoculation (dpi) for the qRT-PCR analysis. The expression analysis of defense-related genes showed that transcript levels of *PATHOGENESIS-RELATED 1* (*PR1*) and *WRKY70* were slightly increased by RKN inoculation, followed by RL treatment, particularly at 7 dpi as compared with that in control plants. However, combined treatment of RL and RKN resulted in a drastic upregulation in the transcript levels of those genes, which peaked at 7 dpi (except for *WRKY70* in roots). For instance, at 7 dpi transcript levels of *PR1* gene were increased by 8-fold and 7-fold, respectively in leaves of RL+RKN plants compared with that of control and RKN only treatment. While transcripts of the *PR1* genes in roots increased gradually until 14 dpi, transcript levels of both *PR1* and *WRKY70* declined after 7 dpi in both roots and leaves of RL+RKN plants. Nonetheless, transcript levels of *PR1* and *WRKY70* in roots of RL+RKN plants still remained elevated at 14 dpi, while those transcripts in leaves declined to the levels of RKN alone in leaves of RL+RKN plants (**Figure 2**).

**FIGURE 3:**
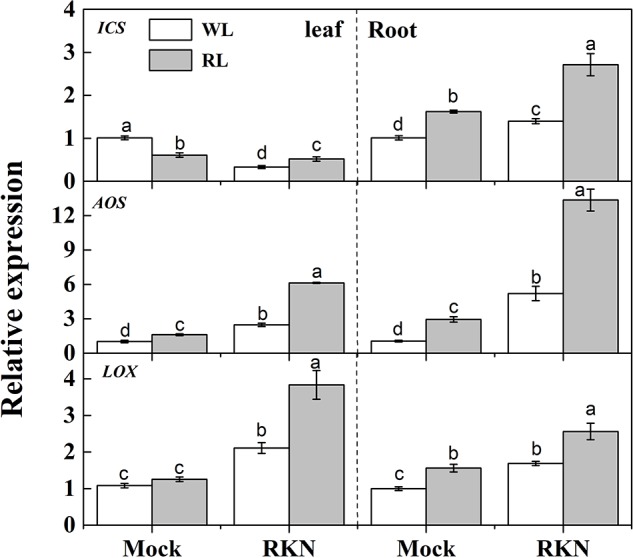
Relative transcript levels of salicylic acid (SA) and jasmonic acid (JA) biosynthetic genes in leaves and roots of watermelon plants. *ICS, LOX*, and *AOS* mRNA abundance was assayed by qRT-PCR at 7 days post inoculation (dpi). Plants were kept in white light (WL, open column) or red light (RL, gray column) without (Mock) or with the inoculation of *M. incognita* (RKN). The primers used for transcript analysis are listed in Supplementary Table S1. Data are the means ±*SD* of three biological replicates. Means denoted by the same letter did not significantly differ at *p* < 0.05 according to Duncan’s multiple range test.

As we noticed a transient peak at 7 dpi for defense gene expression, we then assayed expression of defense hormones-related genes such as SA biosynthetic gene *ISOCHORISMATE SYNTHASE* (*ICS*), and JA biosynthetic gene *ALLENE OXIDE SYNTHASE* (*AOS*) *and LIPOXYGENASE* (*LOX*) at 7 dpi (**Figure 3**). Both RL and RKN single treatment significantly increased transcript levels of those genes over control, except for *ICS* in leaf, which was suppressed by RL or RKN. Consistently, transcripts analysis also showed that combined treatment of RL and RKN resulted in the highest transcript abundance of *ICS, AOS*, and *LOX* at 7 dpi (except for *ICS* in leaf). However, in all tissues tested, RL+RKN increased transcript levels of *ICS, AOS*, and *LOX* at 7 dpi as compared to that in RKN alone (**Figure 3**).

### Red Light Alters Endogenous Hormone Levels in Leaves and Roots

**FIGURE 4:**
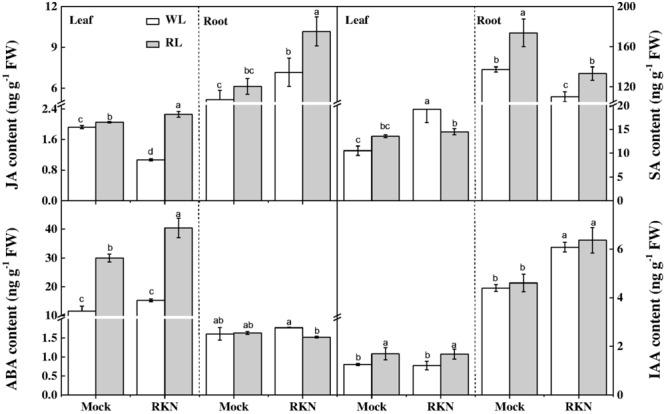
Interactive effects of red light and root knot nematode on endogenous levels of phytohormones in leaves and roots of watermelon plants. SA, JA, IAA, and ABA content was analyzed by LC/MS system at 7 days post inoculation (dpi). Plants were kept in white light (WL, open column) or red light (RL, gray column) without (Mock) or with the inoculation of *M. incognita* (RKN). Data are the means ±*SD* of four replicates. Different letters indicate statistically significant differences (Duncan’s multiple range test with *p* < 0.05).

To further dissect RL and/or RKN-induced changes in endogenous hormone levels, we quantified concentrations of JA, SA, ABA, and IAA in roots and leaves of healthy and infected watermelon plants under RL and white light at 7 dpi. The results showed that contents of IAA, JA, and SA were much higher in roots than that in leaves in all treatments. However, the ABA contents in leaves were approximately 9-fold lower than that of root concentration. As shown in **Figure 4**, RL enhanced JA, ABA, and IAA contents in leaves regardless of RKN treatment. In leaves, while JA content declined, SA content enhanced in response to RKN inoculation. However, RL treatment with RKN inoculation increased JA content but decreased SA content in leaves as compared to that of RKN treatment. Meanwhile, the responses of endogenous hormones to RL or RKN were different in different tissues. In roots, both JA and SA contents were induced by RL+RKN treatment compared to that in RKN alone. ABA content in roots followed the same trend of SA content in leaves, which was not affected by RL in absence of RKN inoculation, but was significantly suppressed by RL in presence of RKN infection. While RKN treatment increased IAA content as compared with mock, RL had no significant effect on IAA content either in mock or RKN treatment (**Figure 4**).

### H_2_O_2_ Is Involved in RL-Mediated Defense Response to RKN

**FIGURE 5:**
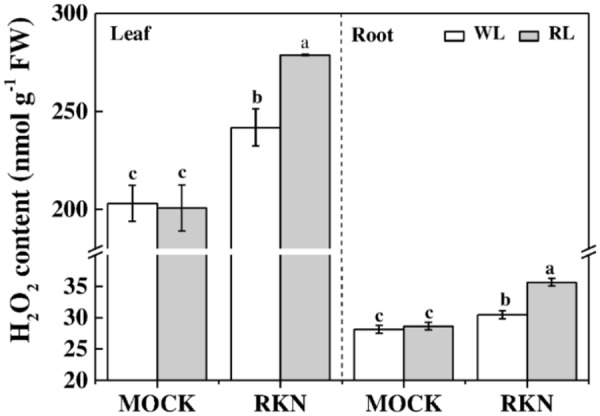
H_2_O_2_ content as affected by red light and/or root knot nematode in leaves and roots of watermelon plants. H_2_O_2_ content was assayed at 7 days post inoculation (dpi) with *M. incognita*. Plants were kept in white light (WL, open column) or red light (RL, gray column) without (Mock) or with the inoculation of *M. incognita* (RKN). Data are the means ±*SD* of four replicates. Different letters indicate statistically significant differences (Duncan’s multiple range test with *p* < 0.05).

H_2_O_2_ is an important molecule that plays dual roles in plant stress response. Therefore, we measured H_2_O_2_ contents in leaves and roots of healthy and infected watermelon plants grown under WL and RL (**Figure 5**). We found that RL alone did not affect the content of H_2_O_2_ both in leaves and roots in absence of RKN infection. However, RKN infection significantly induced H_2_O_2_ levels. More importantly, H_2_O_2_ contents in leaves and roots of RL+RKN plants were significantly higher than that of only RKN plants, indicating a specific signaling role of H_2_O_2_ in RL-promoted defense against RKN.

**FIGURE 6:**
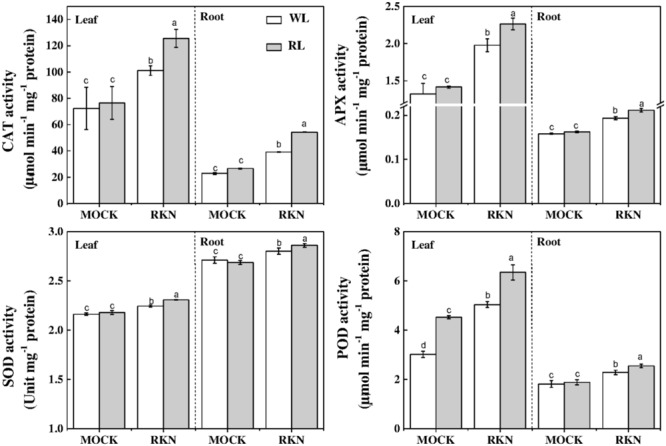
Effects of red light or white light and *M. incognita* infection on the antioxidant enzymes activities in leaves and roots of watermelon. Plants were kept in white light (WL, open column) or red light (RL, gray column) without (Mock) or with the inoculation of *M. incognita* (RKN). The data are the means ± *SD* of four replicates. Different letters indicate statistically significant differences (Duncan’s multiple range test with *p* < 0.05).

To further understand the oxidative status as influenced by RL and/or RKN in plants, we analyzed activities of some key antioxidant enzymes that are responsible for rapid scavenging of ROS. Consistent with the membrane damage as shown in **Figure 2**, the activities of SOD, POD, CAT, and APX were not affected by RL in absence of RKN inoculation. However, RKN infection resulted in a significant increase in the activities of those enzymes both in leaves and roots (**Figure 6**). Interesting, RL+RKN treatment increased all above antioxidant enzyme activities in leaves and roots, compared with that in RKN alone-stressed plants (**Figure 6**).

### Red Light Enhances Defense Against RKN by Stimulating Redox Homeostasis

**FIGURE 7:**
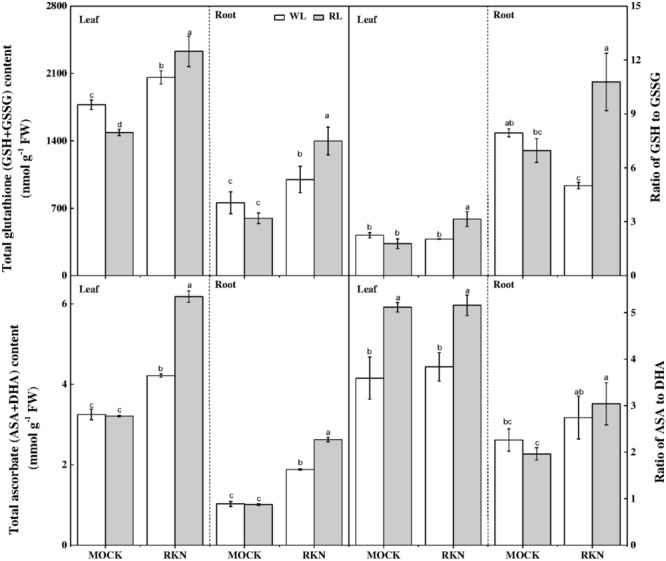
Interactive effects of red light and root knot nematode on ascorbate-glutathione pool in leaves and roots of watermelon plants. Glutathione and ascorbate contents were analyzed spectrophotometrically at 7 days post inoculation (dpi). Plants were kept in white light (WL, open column) or red light (RL, gray column) without (Mock) or with the inoculation of *M. incognita* (RKN). Data are the means ± *SD* of four replicates. Different letters indicate statistically significant differences (Duncan’s multiple range test with *p* < 0.05).

Ascorbate and GSH are the key components of AsA-GSH pools that control cellular redox state and play important role in defense against nematode stress. While total ascorbate remained virtually unchanged in response to RL, RL treatment significantly reduced total GSH content, particularly in leaves, in absence of RKN inoculation (**Figure 7**). However, RKN treatment without RL promoted total AsA and reduced GSH in leaves/roots by 29.73%/83.39%, and 16.02%/31.66%, respectively, compared with mock. Interesting, compared with RKN only treatment, RL treatment on RKN-inoculated plants further increased total AsA and GSH. RL and RKN-induced changes in AsA-GSH pools differentially modulated redox state in leaves and roots. For instance, RL treatment increased AsA:DHA in leaves but not in roots regardless of RKN infection. In line with the trend of total GSH and total ascorbate, RL+RKN significantly increased the ratio of reduced/oxidized glutathione (GSH/GSSG) both in leaves and roots as compared to that in only RKN treatment, indicating a specific effect of RL on redox homeostasis in response to RKN inoculation (**Figure 7**).

## Discussion

Red light stimulates plant defense against both biotrophic and necrotrophic pathogens in a range of plant species (Mutar and Fattah, 2013; Yang et al., 2015b). However, the mechanisms of light quality-mediated defense responses vary from species to species and thus the mechanisms of RL-induced watermelon defense against RKN remain largely unknown. In addition, the roles of diurnal RL exposure of above-ground foliar parts in plant defense against below-ground pathogens remain elusive, particularly in watermelon plants. In this study, we found that the incidence of RKN in watermelon plants was significantly suppressed by diurnal RL compared with that under white light environment. RL-induced enhancement in defense against RKN was closely associated with increased transcript levels of *PRI, WRKY70, ICS, AOS*, and *LOX*, contents of SA, JA, and H_2_O_2_, antioxidant enzyme activity and redox homeostasis in watermelon. This study also suggests that RL improves systemic resistance against RKN through selective regulation of SA and JA biosynthesis in different tissues of watermelon plants.

In the current study, it is highly likely that RL exposure onto watermelon leaves activated systemic resistance against root disease. RL-induced inhibition of nematode development, lipid peroxidation, and electrolyte leakage provide convincing evidence that RL could minimize RKN-induced oxidative stress and damage to watermelon plant. The suppression of RKN disease incidence by RL in the current study is consistent with previous studies in *Arabidopsis* and tomato, where treatment with continuous and nightly RL treatment induced systemic disease resistance against root-knot nematode *M. javanica* and *M. incognita*, respectively (Islam et al., 2008; Yang et al., 2014).

Phytohormones such as SA, JA, ET, IAA, ABA, and BR function as signaling molecules and mediate defense response to phytopathogens (Yang et al., 2015a; Song et al., 2017). In particular, JA and SA play critical roles in plant defense against nematodes including *M. incognita* (Branch et al., 2004; Kammerhofer et al., 2015). In our study, plants inoculated with *M. incognita* accumulated a higher amount of SA, but a lower amount JA in leaves (**Figure 4**). Activation of the SA pathway and suppression of JA pathway is believed to be a strategy by which nematodes antagonizes the host plant immune response for a successful invasion (Ithal et al., 2007; Martìnez-Medina et al., 2017). Thus, our results are consistent with the observations that SA and JA signaling often display inverse patterns of expression in above-ground plant parts (Pieterse et al., 2012). However, JA levels increased in roots upon RKN infection, suggesting that JA might be transported through vascular tissues from above-ground to the below-ground parts and triggered plant defense against nematodes, which resulted in a higher JA content in roots than that in leaves following the RKN infection (Zhang and Baldwin, 1997). We also noticed that RL+RKN treated plants showed an increased JA accumulation both in leaves and roots compared with that of RKN treatment alone (**Figure 4**), which was consistent with the expression of JA biosynthetic genes in respective tissues. Accumulating evidence from previous studies suggests that JA signaling is required for the rhizo-bacteria-induced systemic resistance (Fujimoto et al., 2011). Thus it is highly likely that RL-induced JA biosynthesis perhaps triggers induced systemic resistance (ISR) against RKN in watermelon (Zhang and Baldwin, 1997). Our results are in agreement with the previous reports on tomato plants, which showed that foliar application of methyl jasmonate (MeJA) significantly reduces the infection of RKN (Fujimoto et al., 2011), and the systemic defense signals associated with the JA pathway are transported between above-ground and below-ground plants parts (van Dam et al., 2003). However, SA content in leaves decreased in RL+RKN plants compared with that in RKN alone due to an antagonistic effect of increased JA accumulation in leaves (**Figure 4**).

Unlike leaves, exposure to RL resulted in an increased transcript levels of SA biosynthetic gene *ICS* and elevated SA content in roots after *M. incognita* infection (**Figure 3**), indicating that SA signaling pathway was activated by RL to induce watermelon resistance against nematodes in roots (Nandi et al., 2003; Branch et al., 2004). Notably, SA is also involved in *Mi1*-mediated defense response to root-knot nematode *M. incognita* in tomato (Branch et al., 2004). Meanwhile, JA biosynthetic genes *LOX* and *AOS* as well as JA content were remarkably induced in roots by RL as compared with that in white light condition in response to RKN infection (**Figure 3**), which may indicate a potential synergistic local interaction between SA and JA to combat RKN in roots (van Wees et al., 2000). These results are well in agreement with early findings that RL could stimulate SA and JA biosynthesis and signaling pathway genes to activate plant defense against RKN (van Wees et al., 2000; Yang et al., 2014). This implies that RL-mediated plant defense is attributed to a proper integration of SAR and ISR, which is an interesting mechanism for enhancing plant immunity.

Transcript factor *WRKY70* plays a critical role in resistance (*R*) gene *Mi-1*-mediated resistance against RKN in plants (Atamian et al., 2012). Additionally, *WRKY72* is transcriptionally up-regulated in the roots of resistant tomato genotype and mediates *Mi-1.2*-induced effector-triggered immunity against RKN (Bhattarai et al., 2010). A recent study shows that class-A heat shock factor (*HsfA1a*) and whitefly induced 1 (*Wfi1*)-dependent apoplastic H_2_O_2_ accumulation is required for *Mi-1.2*-mediated resistance against RKN in tomato plants (Zhou et al., 2018). In the current study, we found that RL could induce *WRKY70* transcript and H_2_O_2_ accumulation both in leaves and roots of watermelon plants in presence of RKN inoculation (**Figure 2**), suggesting that WRKY70 and ROS signaling are potentially involved in RL-induced plant defense against RKN in watermelon plants. In addition, tissue specific induction of *WRKY70* may suggest a node of convergence between JA-mediated and SA-mediated signals in plant defense (Li et al., 2004).

A close look into the hormone profiles showed that RL can increase IAA content significantly in the leaf tissues (**Figure 4**), which is consistent with a former research report that RL could trigger phyB-mediated auxin synthesis and increase lower hypocotyl elongation (Guo et al., 2016). Notably, nematode infection also increases root elongation irrespective of white light and RL treatment (Grunewald et al., 2009) as nematode invasion enhances nutrient demand for cell division and differentiation, resulting in giant cell formation at the early stage of nematode infection (Shukla et al., 2017). However, RL treatment on RKN-inoculated plants did not improve IAA content compared with white light treatment on RKN-inoculated plants, indicating that IAA was not involved in RL-induced defense against RKN. In plants, ABA generally plays a negative role in defense against RKN. In the present study, only RKN treatment did not affect ABA content both in leaves and roots, however, RL treatment with RKN inoculation enhanced ABA content in leaves (**Figure 4**). Meanwhile, RL+RKN treatment slightly decreased ABA content compared with that of RKN alone, implying that increased JA biosynthesis might suppress ABA accumulation in roots (Nahar et al., 2012; Kammerhofer et al., 2015). All these results led us to propose that RL-induced watermelon defense against RKN is principally mediated by JA-dependent ISR and SA-dependent SAR, whereas auxin and ABA pathways possibly play minor role in defense against RKN infection.

Hydrogen peroxide (H_2_O_2_) functions as a signaling molecule and mediates plant responses to abiotic and biotic stresses (Neill et al., 2002). In our study, H_2_O_2_ was induced by RL specifically in presence of RKN in watermelon plants (**Figure 5**). This observation supports the assumption that RL could stimulate H_2_O_2_ for defense against pathogens (Wang et al., 2010). Moreover, SA-induced SAR is likely to be mediated by elevated amount of H_2_O_2_ (Chen et al., 1993), indicating a signaling role of H_2_O_2_ in SAR (Alvarez et al., 1998). Therefore, we speculate that RL-induced H_2_O_2_ accumulation in RKN-infected roots might induce H_2_O_2_ in leaves, thus up-regulating the JA signaling pathway genes and JA accumulation (Orozco-Cárdenas et al., 2001).

Both enzymatic and non-enzymatic anti-oxidant systems play an important role in plant tolerance to root-knot nematode *M. incognita*-induced oxidative stress (Oliveira et al., 2012). Here, we also found that the activities of key antioxidant enzymes such as SOD, CAT, and POD and APX were highly induced by RKN (**Figure 6**). Moreover, RL further stimulated antioxidant enzyme and enhanced plant resistance against RKN stress, which is evidenced by reduced MDA content and electrolyte leakage from roots under RL+RKN treatment compared with that in only RKN treatment in watermelon plants. Furthermore, light quality signals, particularly R/FR ratios, are important regulators of antioxidant synthesis and accumulation (Bartoli et al., 2009). R/FR signaling has a major control over the extent of AsA accumulation in leaves over a single photoperiod and JA signaling triggers ascorbate metabolism (Shan and Liang, 2010). In line with this, we noticed a significantly increased AsA content following exposure of watermelon plants to RL+RKN treatment, indicating the important role of RL-induced AsA in enhancing plant defense against RKN. GSH is a key regulator of redox signaling and its buffering activates defense genes (Foyer and Noctor, 2011). Similar to AsA, GSH content was increased by RL treatment in presence of RKN infection, which eventually increased redox homeostasis and enhanced tolerance to RKN. Our findings are in agreement with previous reports that light quality signals regulate the GSH pool (Bartoli et al., 2009) and JA signaling can promote GSH metabolism (Shan and Liang, 2010). Previous reports have also indicated that light quality, especially RL could influence plant tolerance to abiotic and biotic stressors via photoreceptors and phytohormones and photoreceptors such as phytochrome as well (Cerrudo et al., 2012; Yang et al., 2015b; Wang et al., 2016). For instance, *PhyA* and *PhyB* regulate plant tolerance to low temperature stress via ABA-dependent JA signaling in tomato (Wang et al., 2016). Phytochromes also play a critical role in resistance against *Magnaporthe grisea* by regulating SA- and JA-dependent defense pathways in rice plants (Xie et al., 2011). Inactivation of Phy B results in low levels of constitutive defenses and down-regulation of MeJA-induced defenses against herbivore in tomato plants (Cortes et al., 2016). Therefore, in addition to phytohormones, phytochromes may have a role in RL-mediated defense against RKN in watermelon. However, a limitation with a crop like watermelon, versus other model plants, is the lack of targeted mutants in the pathways of interest to establish cause and effect. Therefore, it will be interesting to further explore the role of phytohormones and phytochromes in RL-mediated watermelon defense against RKN by establishing advanced genetic tools for targeted gene suppression in watermelon.

In the current study, we found that RL plays a vital role in plant defense against root knot nematode in watermelon plants. RL acts as a positive signal that triggers tissue specific accumulation of phytohormones and ROS. In addition, RL improves redox homeostasis by modulating the activities of antioxidant enzymes as well as GSH and ASA contents. Our circumstantial evidence suggests that RL exposure on watermelon leaves can enhance systemic defense against RKN infection through a coordinated regulation of SA, JA and redox signaling in watermelon plants. This study provides new insights into the underlying mechanism of RL-induced systemic defense against *M. incognita* in watermelon, which may have potential implication in protected vegetable production.

## Author Contributions

Y-XY, ZY, and JC designed the research. Y-XY andCQWexecuted the experiments. Y-XY, CJW, CPW, and GA analyzed and discussed the data. Y-XY, GA, and JC wrote the manuscript.

## Conflict of Interest Statement

The authors declare that the research was conducted in the absence of any commercial or financial relationships that could be construed as a potential conflict of interest.
