# Supplementary material for: Red Light-Induced Systemic Resistance Against Root-Knot Nematode Is Mediated by a Coordinated Regulation of Salicylic Acid, Jasmonic Acid and Redox Signaling in Watermelon
Source: Front Plant Sci. 2018 Jul 10;9:899. doi: 10.3389/fpls.2018.00899 (PMC6048386; doi:10.3389/fpls.2018.00899)
Supplement: Supplementary file 1 [file Table_1.DOCX]

**Supplementary Table S1: Primers used for real time qPCR assays**

| **Gene ID** | **Functional annotation** | **Forward Primer** | **Reverse Primer** |
| --- | --- | --- | --- |
| Cla020794 | clathrin adaptor complex subunit (CAC) | AATTGTGGTTGATGCTGCAC | TGACAGCTGTACCTGGCATC |
| Cla003129 | α-tubulin (TUA) | CTTGCTGGGAGCTCTATTGC | AACGGATTAAAAGCGTCGTG |
| Cla001621 | Pathogensis-related 1  (PR1) | TCCTCAATGCCCACAACGCT | AGGTCCGACACCAACTTGGGCACGAGC |
| Cla007307 | *WRKY transcription factor 70*  (*WRKY70* ) | AGCTCTTGCCACAAAGATCTTGAC | GTGAGTCGCCGGAATCCAGA |
| Cla022526 | *Allene oxide synthase*  (*AOS*) | AACCGTCGGCGATTGAG | GGGAAGTGATTTGTGGGTAT |
| Cla019128 | *Isochorismate synthase*  (*ICS*) | CGGCAGAAGCAGAGCCATTG | CAATGAAGGCGGCGGAAGAC |
| Cla014154 | *Lipoxygenase*  (*LOX*) | ATGCTATGGTTGACACGATTTG | CTTATGTGAACCACTATTACCCAGA |
