# Supplementary material for: Red Light-Induced Systemic Resistance Against Root-Knot Nematode Is Mediated by a Coordinated Regulation of Salicylic Acid, Jasmonic Acid and Redox Signaling in Watermelon
Source: Front Plant Sci. 2018 Jul 10;9:899. doi: 10.3389/fpls.2018.00899 (PMC6048386; doi:10.3389/fpls.2018.00899)
Supplement: Supplementary file 2 [file Table_2.DOCX]

**Supplementary Table S2. Parameters used for detection of phytohormones and related compounds by LC-MS/MS.**

| **Compound** | **Capillary**  **CID^1^**  **(V)** | **Molecular ion [M-H] (*m/z*)** | **Fragment ion**  **(*m/z*)** | **CE^2^**  **(V)** | **Reference** |
| --- | --- | --- | --- | --- | --- |
| JA | 50 | 209.1 | 59.1 | 2 | Wu *et al*. (2007) |
| D_5_-JA (IS) | 116 | 214.3 | 62.1 | 8 | Alba *et al*. (2015) |
| SA | 75 | 137 | 93 | 10 | Wu *et al*. (2007) |
| D_4_-SA (IS) | 80 | 141 | 97 | 16 | Wu *et al*. (2007) |
| ABA | 75 | 263.1 | 153 | 0 | Durgbanshi *et al*. (2005) |
| D_6_-ABA (IS) | 162 | 269.3 | 159.2 | 0 | Durgbanshi *et al*. (2005) |
| IAA | 75 | 176.1 | 130.1 | 10 | Durgbanshi *et al*. (2005) |
| D_5_-IAA(IS) | 70 | 181.2 | 134.2 | 12 | Boelaert *et al*. (2013) |

^1^collision-induced dissociation; ^2^collision energy; IS, internal standard.
